# Supplementary material for: Selective control of conductance modes in multi-terminal Josephson junctions
Source: Nat Commun. 2022 Oct 8;13:5933. doi: 10.1038/s41467-022-33682-2 (PMC9547902; doi:10.1038/s41467-022-33682-2)
Supplement: Supplementary file 1 — Supplementary Information [file 41467_2022_33682_MOESM1_ESM.pdf]

Supplementary Information for "Selective Control of Conductance Modes in Multi-terminal Josephson Junctions"

Gino V. Graziano,[1,†] Mohit Gupta,[1,†] Mihir Pendharkar,[2,3] Jason T. Dong[4],
Connor P. Dempsey,[2] Chris Palmstrøm,[2,4,5] and Vlad S. Pribiag[1*]

[1]School of Physics and Astronomy, University of Minnesota, Minneapolis, Minnesota 55455, USA

[2]Electrical and Computer Engineering, University of California Santa Barbara, Santa Barbara, California 93106, USA

[3]Materials Science and Engineering, Stanford University, Stanford, California 94305, USA

[4] Materials Department, University of California Santa Barbara, Santa Barbara, California 93106, USA

[5] California NanoSystems Institute, University of California Santa Barbara, Santa Barbara, California 93106, USA

† These authors contributed equally: Gino V. Graziano and Mohit Gupta.

*To whom correspondence should be addressed; E-mail: vpribiag@umn.edu.

I. HALL MEASUREMENTS

We have constructed a Hall bar device to measure the mean free path of the 2DEG. Shubnikov-de Hass (SdH) oscillations in the longitudinal resistance (R_{xx}) are observed. By taking a linear fit of the inverse values of magnetic flux densities where maxima of Sdh oscillations occur, the charge density n can be evaluated as:

$$n = \frac{2e}{mh} \tag{1}$$

where m is the slope of the linear fit. From our measurements we get $m = 0.04\mathrm{T}^{-1}$, this

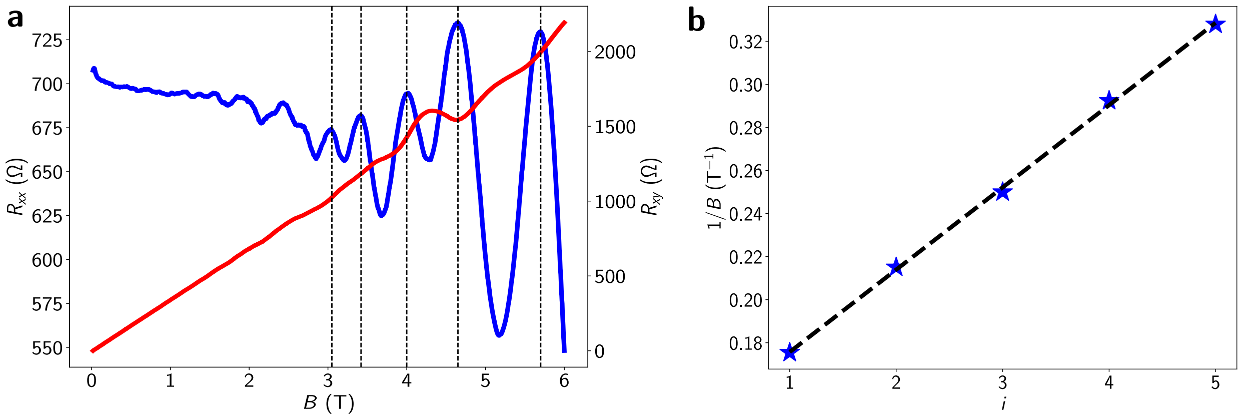

Supplementary Fig. 1. **a** Symmetrized longitudinal resistance R_{xx} (shown in blue) and anti-symmetrized transverse resistance R_{xy} (shown in red) as a function of magnetic field of the Hall bar device. To extract the period of SdH oscillations the peaks in the resistances are highlighted by dashed black lines. **b** Inverse of magnetic field values as a function of maxima of SdH oscillations (blue stars). Linear fit is shown by dashed black line.

gives a charge density of $n = 1.22 \times 10^{12}$ cm^{-2}. The mobility can be evaluated by:

$$\mu = \frac{1}{en\rho_{xx}(B=0)} \tag{2}$$

$\rho_{xx} = R_{xx}(W/L)$, here W is the channel width of the constructed Hall bar and L is distance between he probes used to measure R_{xx}. From our measurements we get $\rho_{xx}(B=0) = 515$ Ω. This give a mobility of $\mu = 9920$ cm^{2}V^{-1}s^{-1}. The mean free path, ℓ can be evaluated by:

$$\ell = \frac{\hbar}{e}\mu\sqrt{2\pi n} \tag{3}$$

Using the above mentioned μ and n we get $\ell \sim 180$ nm.

II. DIFFERENTIAL RESISTANCE MAP IN VOLTAGE SPACE

Differential resistance maps for Device 1 are plotted in V_1-V_2 space. The lower resistance feature along $V_1 = -V_2$ which can be attributed to Cooper quartet transport is clearly visible.

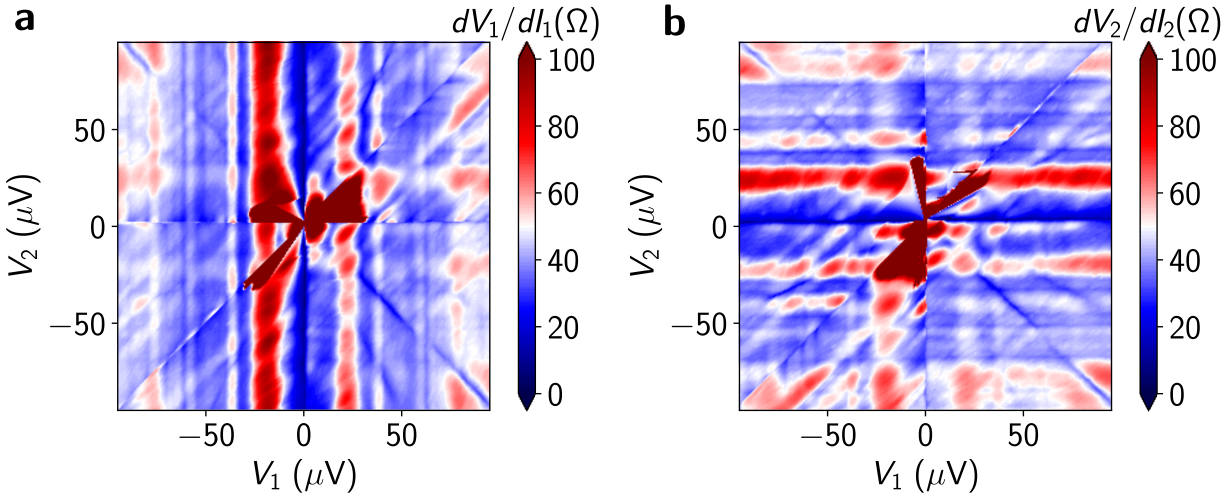

Supplementary Fig. 2. **a** Measurement of dV_1/dI_1 on Device 1 at small magnetic field **b** Measurement of dV_2/dI_2 on Device 1 at small magnetic field.

III. RESISTOR NETWORK MODEL

For deducing the behavior of the system under two independent current or voltage biases, we can treat the trijunction as a Δ-type resistor network. The Δ-type resistor network consists simply of three nodes $\{1, 2, 0\}$ with resistors $\{R_1, R_2, R_3\}$ connecting each pair. In the context of the real trijunction device: the nodes correspond to the three aluminum contacts, and when the system is driven into a completely resistive state, the values of the network resistances correspond to the pairwise normal state resistances $R_{\mathrm{n},i}$ of the Josephson junctions between the terminals.

The transformation from Δ to Y yields the following expressions for the resistances in the Y-equivalent network:

$$R'_1 = \frac{R_1 R_3}{R_1 + R_2 + R_3},\ R'_2 = \frac{R_2 R_3}{R_1 + R_2 + R_3},\ R'_3 = \frac{R_1 R_2}{R_1 + R_2 + R_3} \tag{4}$$

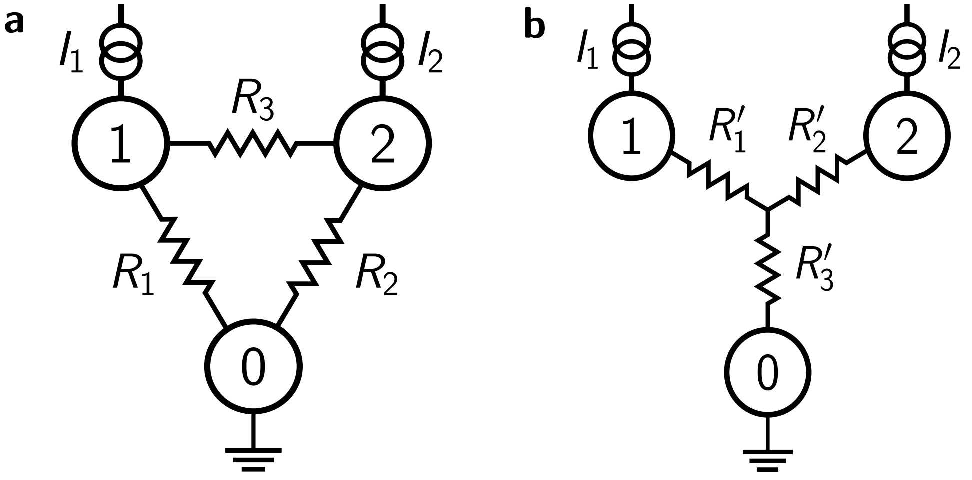

Supplementary Fig. 3. **a** Δ network **b** Equivalent Y network.

Applying Kirchoff's laws to the Y network with node 0 grounded ($V_0 = 0$) yields:

$$V_1 = I_1(R'_1 + R'_3) + I_2R'_3,\ V_2 = I_1R'_3 + I_2(R'_2 + R'_3),\ V_1 - V_2 = I_1R'_1 - I_2R'_2 \tag{5}$$

Using these equations, we can set V_1, V_2 or $V_1 - V_2$ equal to 0 to identify the relations between I_1 and I_2 that yield zero voltage between terminals. The superconducting arm features of the 2D resistance map figures will be centered along these lines.

$$V_1 = 0 \to I_2 = -\left(\frac{R_3}{R_2} + 1\right) I_1,\ V_2 = 0 \to I_2 = -\left(\frac{R_3}{R_1} + 1\right)^{-1} I_1,\ V_1 - V_2 = 0 \to I_2 = \frac{R_1}{R_2} I_1 \tag{6}$$

For a fully symmetric junction ($R_1, R_2, R_3 = R$) we recover that the superconducting features lie along the lines $I_2 = -2I_1$, $I_2 = -\frac{1}{2}I_1$ and $I_1 = I_2$.

For selective gating, where one leg of the junction is under the effect of a more negative gate voltage, we can study the behavior of the superconducting feature lines by looking at the limits of equations (3), (4) and (5). Locally negatively gating one leg relative to the others will ideally increase the resistance between the two terminals that most closely span the leg.

Depleting the leg between terminals 1 and 2 increases R_3. Taking the limit as $R_3 \to \infty$:

$$V_1 = 0 \to I_1 = 0,\ V_2 = 0 \to I_2 = 0,\ V_1 - V_2 = 0 \to I_1 = \frac{R_1}{R_2} I_1 \text{ (Unchanged)} \tag{7}$$

Depleting the leg between terminals 1 and 0 increases R_1. Taking the limit as $R_1 \to \infty$:

$$V_1 = 0 \rightarrow I_2 = -\left(\frac{R_3}{R_2} + 1\right) I_1 \text{ (Unchanged)},\ V_2 = 0 \rightarrow I_2 = -I_1,\ V_1 - V_2 = 0 \rightarrow I_1 = 0 \quad (8)$$

Depleting the leg between terminals 2 and 0 increases R_2. Taking the limit as $R_2 \rightarrow \infty$:

$$V_1 = 0 \rightarrow I_2 = -I_1,\ V_2 = 0 \rightarrow I_2 = -\left(\frac{R_3}{R_1} + 1\right)^{-1} I_1 \text{ (Unchanged)},\ V_1 - V_2 = 0 \rightarrow I_2 = 0 \quad (9)$$

IV. RCSJ SIMULATION

To model the behavior of a device with preferential gating, we simulated a three-terminal Josephson junction using an resistively and capacitatively shunted junction (RCSJ) network model. The simulation considers three nodes with three RCSJ's connecting each pair. These RCSJs between any two superconducting nodes i, j, have three parameters consisting of their critical current $I_{c,ij}$, normal state resistance $R_{n,ij}$ and capacitance C_{ij}. All the capacitances ($C_{i,j}$) are set to $C = 5\times10^{-15}$ F. This small capacitance is only necessary for the stabilization of the simulations. The current between any two nodes is then:

$$I_{ij} = I_{c,ij}\sin(\phi_{ij}) + (\frac{1}{R_{n,ij}})\frac{\hbar}{2e}\dot{\phi}_{ij} + C_{ij}\frac{\hbar}{2e}\ddot{\phi}_{ij} \quad (10)$$

Here ϕ_{ij} is the superconducting phase difference between i and j node. Due to gauge invariance we can set node 0's phase $\phi_0 = 0$, this allows the notation to be simplified, with $\phi_{10} = \phi_1$, $\phi_{20} = \phi_2$ and $\phi_{12} = \phi_1 - \phi_2$. We simplify also $R_{n,10} = R_{n,1}$, $R_{n,20} = R_{n,2}$ and $R_{n,12} = R_{n,3}$. Using Kirchhoff's law at two node 1 and 2 we get the following two equations:

$$\begin{aligned} I_1 &= I_{c,1}\sin(\phi_1) + (G_{n,1} + G_{n,3})\frac{\hbar}{2e}\dot{\phi}_1 + 2C\frac{\hbar}{2e}\ddot{\phi}_1 \\ &+ I_{c,3}\sin(\phi_1 - \phi_2) - G_{n,3}\frac{\hbar}{2e}\dot{\phi}_2 - C\frac{\hbar}{2e}\ddot{\phi}_2 \end{aligned} \quad (11)$$

$$\begin{aligned} I_2 &= I_{c,2}\sin(\phi_2) + (G_{n,2} + G_{n,3})\frac{\hbar}{2e}\dot{\phi}_2 + 2C\frac{\hbar}{2e}\ddot{\phi}_2 \\ &- I_{c,3}\sin(\phi_1 - \phi_2) - G_{n,3}\frac{\hbar}{2e}\dot{\phi}_1 - C\frac{\hbar}{2e}\ddot{\phi}_1 \end{aligned} \quad (12)$$

here $G_{n,i} = 1/R_{n,i}$ is the conductance between any two pairs of terminals. One can rewrite Eq. 12 for $\ddot{\phi}_1$ and $\ddot{\phi}_2$. This leads to a second order differential equation for the phase variable

Figure	$I_{c,1}$	$I_{c,2}$	$I_{c,3}$	$R_{n,1}$	$R_{n,2}$	$R_{n,3}$
Figure 3**d** (main text)	180 nA	130 nA	30 nA	220 Ω	280 Ω	600 Ω
Supplementary Fig. 4**c**	350 nA	50 nA	350 nA	125 Ω	425 Ω	125 Ω
Supplementary Fig. 4**f**	125 nA	325 nA	450 nA	242 Ω	110 Ω	110 Ω

Supplementary Table I. Table of parameter values used to produce plots in Supplementary Fig. 4

$\Phi = \begin{pmatrix} \phi_1 \\ \phi_2 \end{pmatrix}$:

$$\frac{\hbar}{2e}\mathcal{C}\ddot{\Phi} + \frac{\hbar}{2e}\mathcal{G}\dot{\Phi} = \mathcal{I} - f(\Phi) \quad (13)$$

with:

$$\mathcal{C} = \begin{pmatrix} 2C & -C \\ -C & 2C \end{pmatrix} \quad (14)$$

$$\mathcal{G} = \begin{pmatrix} G_{n,1} + G_{n,3} & -G_{n,3} \\ -G_{n,3} & G_{n,2} + G_{n,3} \end{pmatrix} \quad (15)$$

$$\mathcal{I} = \begin{pmatrix} I_1 \\ I_2 \end{pmatrix} \quad (16)$$

$$f(\Phi) = \begin{pmatrix} I_{c,1}\sin(\phi_1) + I_{c,3}\sin(\phi_1 - \phi_2) \\ I_{c,2}\sin(\phi_2) - I_{c,3}\sin(\phi_1 - \phi_2) \end{pmatrix} \quad (17)$$

The above second order differential equation can be solved numerically to find $\Phi(t)$ for given initial conditions and $\langle\dot{\Phi}\rangle$ can be calculated this allows us to get the two voltage drops $V_1 = \langle\dot{\phi_1}\rangle$ and $V_2 = \langle\dot{\phi_2}\rangle$ for a given value of I_1 and I_2. We utilize the approach outlined in the Ref. [1] using Pytorch library to solve in parallel for a grid of I_1 and I_2 allowing for rapid computation. Source code for the performed simulations is provided with this manuscript.

Supplementary Fig. 4**a** and 4**d** shows schematics of preferential gating along the two junction legs not included in the main text. The $I_{c,i}$ and $R_{n,i}$ values in the simulation were tuned by iteration to match the experimental data (Supplementary Fig. 4**b** and **e**) taken in these configurations. The values used in the presented simulation figures (Figure. 3**d** in main text and Supplementary Fig. 4**c**, **f**) can be found in Supplementary Table I.

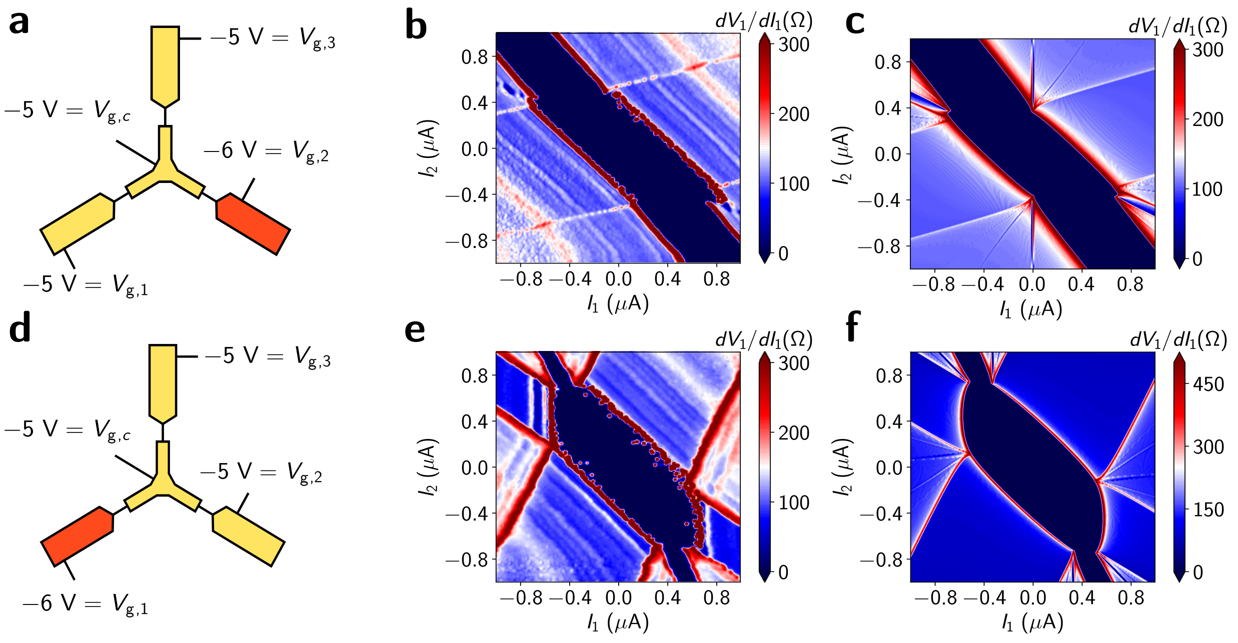

Supplementary Fig. 4. **a** Schematic of gate configuration for the gating of junction leg between terminal 0 and 2. **b** Measurement of dV_1/dI_1 at $V_{g,c}, V_{g,1}, V_{g,3} = -5$V and $V_{g,2} = -6$ V. **c** RCSJ simulation of dV_1/dI_1 with parameters tuned to match the features of experimental data in **b**. **d** Schematic of gate configuration for the gating of junction leg between terminal 0 and 1. **e** Measurement of dV_1/dI_1 at $V_{g,c}, V_{g,2}, V_{g,3} = -5$V and $V_{g,1} = -6$ V. **f** RCSJ simulation of dV_1/dI_1 with parameters tuned to match the features of experimental data in **e**.

V. CONDUCTANCE DATA FROM DEVICE 2 AND DEVICE 3

Conductance data for Device 2 and Device 3 showing accessibility of single mode regime coexistent with superconductivity in all three legs of the devices are shown in Supplementary Fig. 5 **a,b,c** and Supplementary Fig. 6 **a,b,c**, respectively. For Device 3 we also observe structures similar to Coulomb diamonds for high negative gate voltage. Data measured at elevated temperature of 2.1 K and at out-of plane magnetic field of $B = 0.95$T for the Device 2 smooths out conductance data (Supplymentry Fig. 5 e, f), due to suppression of coherent backscattering.

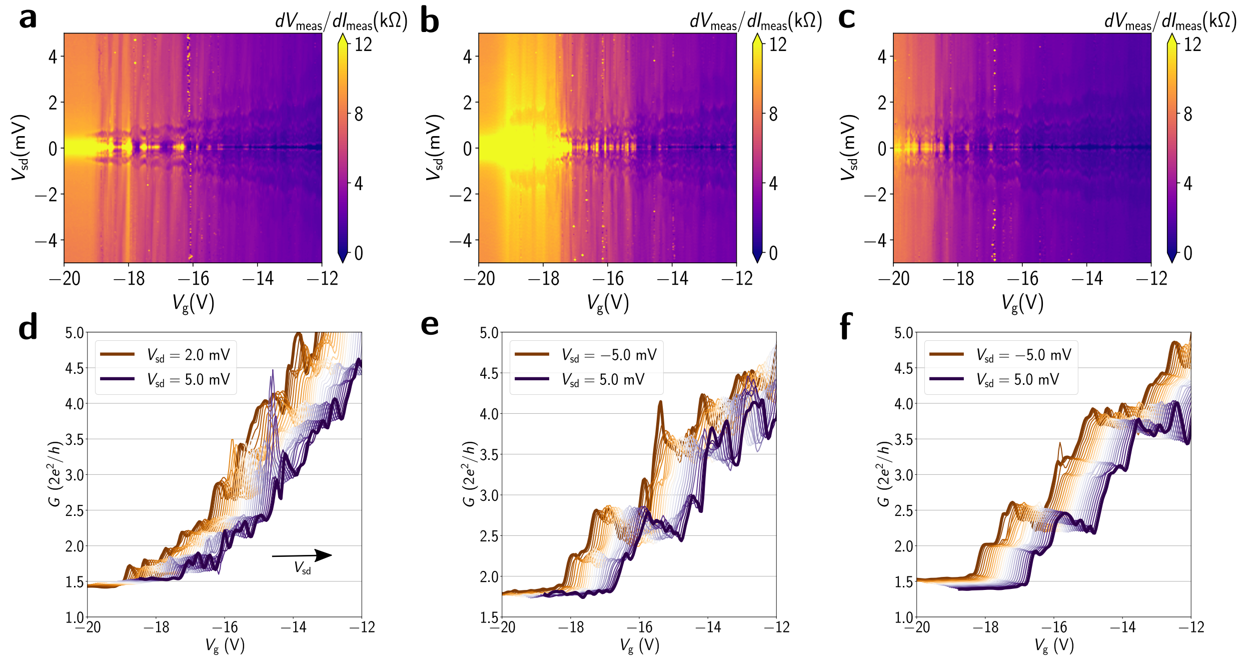

Supplementary Fig. 5. Color map of differential resistance as a function of source-drain bias $\mathrm{V_{sd}}$ and gate voltage $\mathrm{V_g}$ for Device 2 at $B = 0$ and $T = 90$ mK **a** for terminal pair 0 and 1, **b** terminal pair 0 and 2 and **c** terminal pair 1 and 2. Differential conductance as a function of gate voltage for different V_{sd} for Device 2 at **d** $B = 0$ and $T = 90$ mK **e** at $B = 0$ and $T = 2$ K **f** at $B = 0.95$ T and $T = 90$ mK for terminal pair 0 and 1. The curves correspond to increments in V_{sd} of 0.125 mV, and are offset on the gate voltage (arrow indicating direction of increasing V_{sd}) by 3 mV for clarity. The V_{sd} range is shown in plot legends.

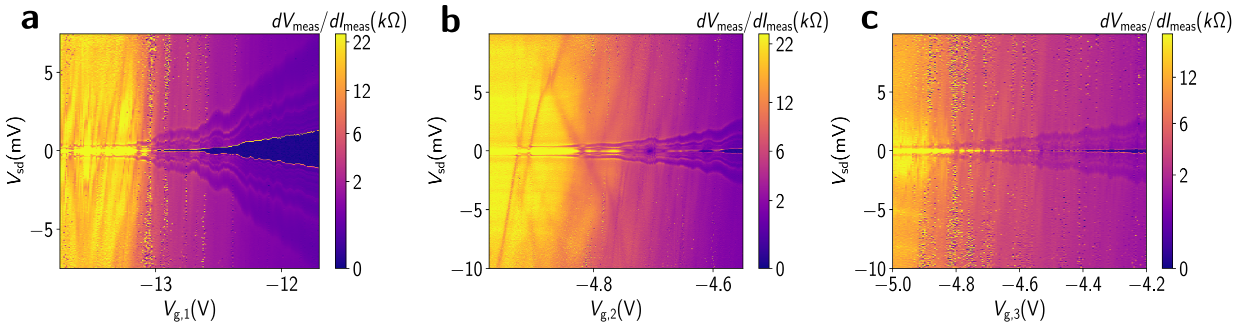

Supplementary Fig. 6. Color map of differential resistance as a function of source-drain bias V_{sd} and split gate voltages for Device 3 at $B = 0$ and $T = 50$ mK for **a** terminal pair 0 and 1 at $V_{g,2} = -5.1$ V and $V_{g,3} = -7.5$ V while $V_{g,1}$ is swept. **b** terminal pair 0 and 2 at $V_{g,1} = -6$ V and $V_{g,3} = -3$ V while $V_{g,2}$ is swept and **c** terminal pair 1 and 2 at $V_{g,1} = -7$ V and $V_{g,2} = -7$ V while $V_{g,3}$ is swept.

VI. DATA FROM DEVICE 4

Here we present data from a fourth Device lithographically identical to Device 1 and Device 3. We see similar transport features, showing a high degree of reproducibility of such devices.

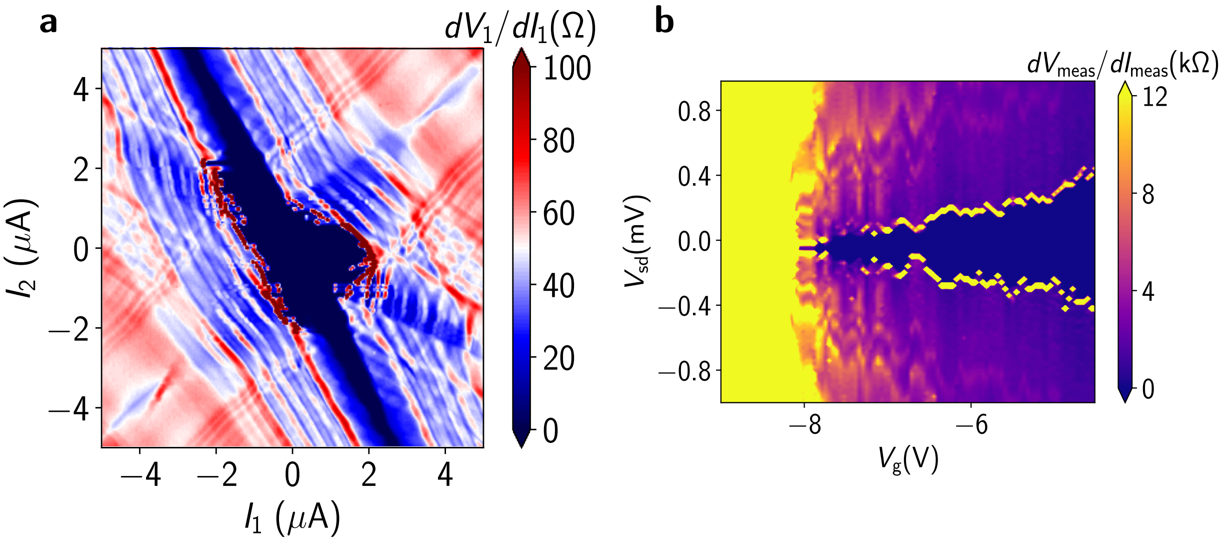

Supplementary Fig. 7. **a** Measurement of dV_1/dI_1 on Device 4 at small magnetic field and $T = 30$ mK. **b** Differential conductance as a function of gate voltage and V_{sd} for Device 4 at small magnetic field and $T = 30$ mK

SUPPLEMENTARY REFERENCES

[1] Arnault, E. G. *et al.* Dynamical stabilization of multiplet supercurrents in multiterminal josephson junctions. *Nano Letters* **22**, 7073–7079 (2022).
